# Hyperoxia effects on intensive care unit mortality: a retrospective pragmatic cohort study

**DOI:** 10.1186/s13054-018-2142-6

**Published:** 2018-09-21

**Authors:** Mathilde Ruggiu, Nadia Aissaoui, Julien Nael, Caroline Haw-Berlemont, Bertrand Herrmann, Jean-Loup Augy, Sofia Ortuno, Damien Vimpère, Jean-Luc Diehl, Clotilde Bailleul, Emmanuel Guerot

**Affiliations:** 1 Department of Intensive Care, Hôpital européen Georges Pompidou, Assistance Publique des Hôpitaux de Paris, 20 rue Leblanc, 75015 Paris, France; 2 Université Paris Descartes, 12 rue de l’école de Médecine, 75006 Paris, France; 3 INSERM U970, 20 rue Leblanc, 75015 Paris, France

Supplementary oxygen is frequent in the management of patients admitted to the intensive care unit (ICU) [1]. However, some studies have suggested deleterious effects of hyperoxia on these patients [2–4]. This main study objective was to assess the association between hyperoxia, at any time of the ICU stay, and ICU mortality regardless of the cause of patient admission.

Our study was an observational, retrospective, and single-centre study in the Hôpital Européen George Pompidou medical ICU, Paris, France. All patients admitted between November and December 2017 were included regardless of their admission cause and all of their arterial blood gases (ABGs) were analysed. Hyperoxia was defined as a partial arterial pressure in oxygen (PaO_2_) superior to 100 mmHg (13.3 kPa). The principal judgement criterion was occurrence of at least one hyperoxia episode during the ICU stay. All statistical tests were two-tailed with a significance threshold of 0.05. Analyses were performed with R v3.2.4. Survival analysis was estimated by Kaplan–Meier methods.

A total of 130 patients, median age 68 (57–79) years and median SAPS II 45 (35–56), were included. The mean reason for ICU admission was respiratory failure (60 patients, 46%) and 83 patients (64%) needed mechanical ventilation. Thirty-five patients (27%) died during their ICU stay.

Eighty patients (62%) presented at least one episode of hyperoxia. Overall survival (OS) was significantly lower in patients who presented at least one episode of hyperoxia during their ICU stay: median OS was 26 days (95% CI 20–NR) versus median not reached, *p* = 0.0047 (Fig. 1).

**Fig. 1:**
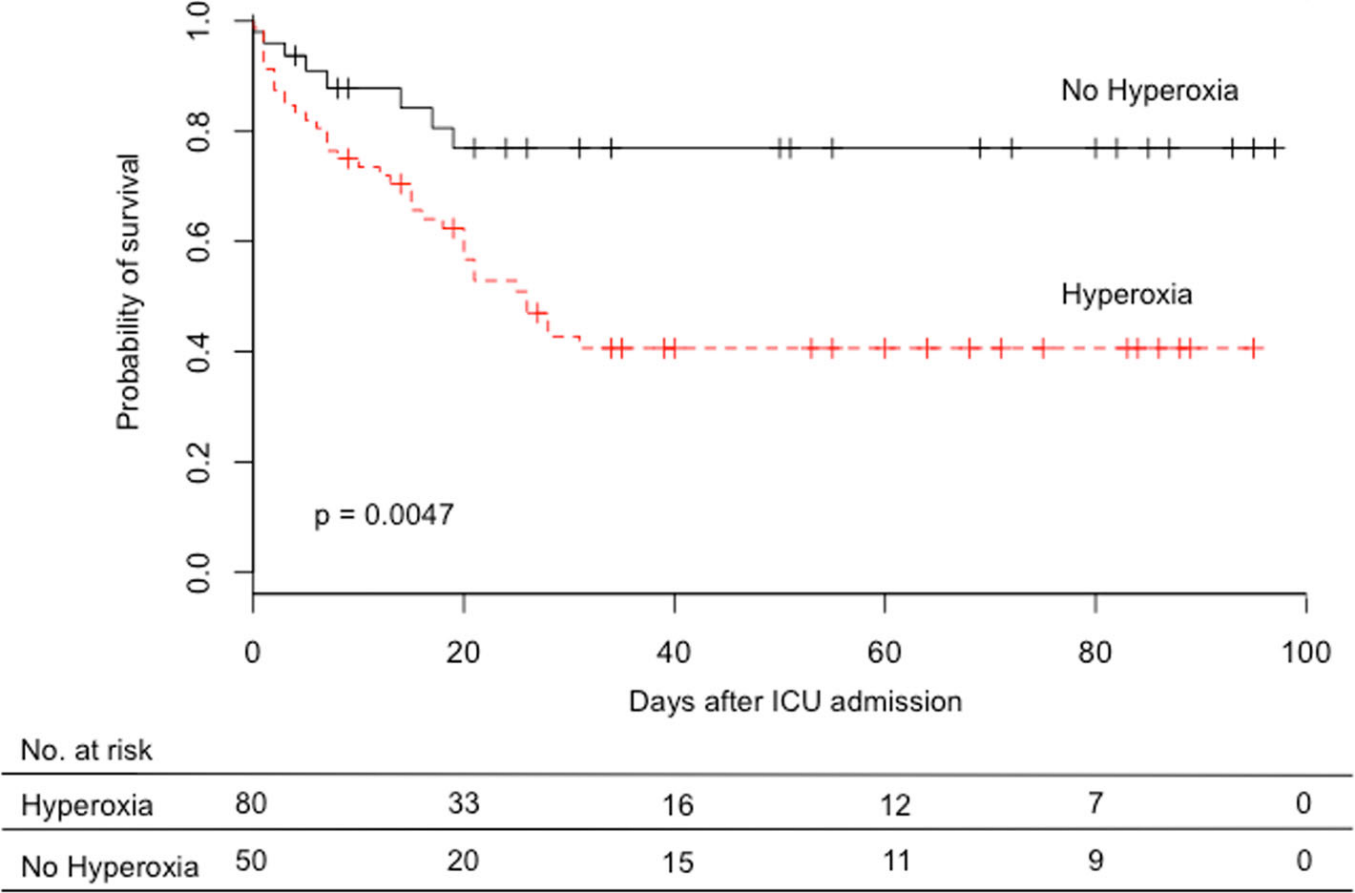
Kaplan–Meier estimation of overall survival. Hyperoxia group, patients who presented at least one hyperoxia episode during ICU stay. No hyperoxia group, patients who presented no hyperoxia episode during ICU stay. Overall survival median in hyperoxia group 26 days (95% CI 20–NR); overall survival median in no hyperoxia group not reached. ICU intensive care unit

In univariate analysis, hyperoxia was a risk factor for mortality: 31 deceased patients presented hyperoxia (89%) versus 49 alive patients (52%), *p* < 0.001. SAPS II and mechanical ventilation time were also mortality risk factors in univariate analysis (Additional file 1: Table S1). Multivariate analysis showed that hyperoxia was an independent risk factor for ICU mortality: OR = 3.80 (95% CI 1.08–16.01), *p* = 0.047 (Table 1).

**Table 1:** Mortality risk factors in the medical ICU, multivariate analysis

Mortality risk factor	Deceased (*n* = 35)	Alive (*n* = 95)	Odds ratio (95% CI)	*p*
Age (years)	70 (66–75)	65 (61–69)		0.15
SAPS II	64 (56–72)	41 (37–45)		< 0.001
Mechanical ventilation time	9 (7–12)	5 (3–7)		0.074
At least one PaO_2_ > 100 mmHg (13.3 kPa)	31 (89%)	49 (52%)	3.80 (1.08–16.01)	0.047

Data presented as mean (95CI) or *n* (%)

*R*^2^ = 0.453

*CI* confidence interval, *ICU* intensive care unit, *PaO*_*2*_ partial arterial pressure in oxygen, *SAPS* II Simplified Acute Physiology Score II

Despite a conservative oxygen policy, 62% of patients presented at least one episode of hyperoxia, which reinforces the statement by Helmerhorst et al. [5]. Previous studies had a focus on specific categories of selected patients [2–4]. On the contrary, our study is a pragmatic study in real-life conditions. We included all consecutive patients admitted to the medical ICU without any exclusion criteria, regardless of the admission cause, mechanical ventilation need, or initial severity, and we collected prospectively a large amount of 1.450 ABG. In this study, we demonstrated that hyperoxia at any time of the ICU stay significantly decreases OS and is an independent mortality risk factor.

## Additional file

Additional file 1: **Table S1.** Mortality risk factors in medical ICU, univariate analysis. (DOCX 17 kb)

## Abbreviations

ABG: Arterial blood gas
ICU: Intensive care unit
OS: Overall survival
PaO_2_: Partial arterial pressure in oxygen
SAPS II: Simplified Acute Physiology Score II

## References

[1] Panwar R, Capellier G, Schmutz N, Davies A, Cooper DJ, Bailey M (2013). Current oxygenation practice in ventilated patients—an observational cohort study. Anaesth Intensive Care.

[2] Vincent J-L, Taccone FS, He X (2017). Harmful effects of hyperoxia in postcardiac arrest, sepsis, traumatic brain injury, or stroke: the importance of individualized oxygen therapy in critically ill patients. Can Respir J.

[3] Damiani E, Adrario E, Girardis M, Romano R, Pelaia P, Singer M, et al. Arterial hyperoxia and mortality in critically ill patients: a systematic review and meta-analysis. Crit Care [Internet]. 2014;4(1):23. Available from: http://ccforum.biomedcentral.com/articles/10.1186/s13054-014-0711-x. Cited 2018 Jul 15. 10.1186/s13054-014-0711-x PMC4298955 25532567

[4] Helmerhorst HJF, Roos-Blom M-J, van Westerloo DJ, de Jonge E (2015). Association between arterial hyperoxia and outcome in subsets of critical illness: a systematic review, meta-analysis, and meta-regression of cohort studies. Crit Care Med.

[5] Helmerhorst HJ, Schultz MJ, van der Voort PH, Bosman RJ, Juffermans NP, de Jonge E, et al. Self-reported attitudes versus actual practice of oxygen therapy by ICU physicians and nurses. Ann Intensive Care [Internet]. 2014;18(6):711. Available from: http://www.annalsofintensivecare.com/content/4/1/23. Cited 2018 Jul 15. 10.1186/s13613-014-0023-y PMC4240734 25512878

