# Supplementary material for: Hyperoxia effects on intensive care unit mortality: a retrospective pragmatic cohort study
Source: Crit Care. 2018 Sep 21;22:218. doi: 10.1186/s13054-018-2142-6 (PMC6148961; doi:10.1186/s13054-018-2142-6)
Supplement: Supplementary file 1 — Table S1. Mortality risk factors in medical ICU, univariate analysis. (DOCX 17 kb) [file 13054_2018_2142_MOESM1_ESM.docx]

Additional file 1: Table S1: Mortality risk factor in medical ICU, univariate analysis

| Mortality risk factor | Deceased  (n=35) | Alive  (n=95) | *p* |
| --- | --- | --- | --- |
| Age (years) | 70 [66 – 75] | 65 [61 – 69] | 0.056 |
| Male sex | 23 (66%) | 62 (65%) | 0.96 |
| SAPS II | 64 [56 – 72] | 41 [37 – 45] | <0.001 |
| Admission of respiratory disease | 18 (51%) | 42 (44%) | 0.46 |
| Hyperoxia parameters |  |  |  |
| - At least one PaO2 > 100 mmHg | 31 (89%) | 49 (52%) | <0.001 |
| - Time in hyperoxia (% of stay) | 28 [19-38] | 17 [12- 23] | 0.042 |
| - Median PaO2 (mmHg / kPa) | 89 [80-98]  11.9 [10.7-13.1] | 85 [80-91]  11.3 [10.7-12.1] | 0.35 |
| - Maximal PaO2 _95_CI (mmHg / kPa) | 173 [146-200]  23.1 [19.5-26.7] | 118 [107-130]  15.7 [14.3-17.3] | <0.001 |
| - % PaO2 > 100 mmHg | 31 [21 – 40]  [13.3 kPa] | 23 [16 – 29] | 0.16 |
| Mechanical ventilation time (days) | 9 [7 – 12] | 5 [3 – 7] | 0.01 |
| Quantitative variables are expressed as mean, [_95_CI] and qualitative variables are expressed as number (%)  Abbreviations: ICU = intensive care unit, O2 = oxygen, PaO2 = partial arterial pressure in oxygen, SAPS II = Simplified Acute Physiology Score II | | | |
